# Influence of osteogenic stimulation and VEGF treatment on in vivo bone formation in hMSC-seeded cancellous bone scaffolds

**DOI:** 10.1186/1471-2474-15-350

**Published:** 2014-10-16

**Authors:** Ulrich Lenze, Florian Pohlig, Sebastian Seitz, Christina Ern, Stefan Milz, Denitsa Docheva, Matthias Schieker

**Affiliations:** Laboratory of Experimental Surgery and Regenerative Medicine, Department of Surgery, University of Munich (LMU), Munich, Germany; Department of Orthopedics and Orthopedic Sports Medicine, Technical University of Munich (TU), Munich, Germany; Department of Trauma Surgery, Technical University of Munich (TU), Munich, Germany; Department of Orthopaedics, University Medical Center Hamburg-Eppendorf, Hamburg, Germany; Department of Restorative Dentistry & Periodontology, University of Munich (LMU), Munich, Germany; Department of Anatomy, University of Munich (LMU), Munich, Germany

**Keywords:** hMSC, Tissue engineering, VEGF, Vascularization, Osteogenic stimulation

## Abstract

**Background:**

Tissue engineering approaches for reconstruction of large bone defects are still technically immature, especially in regard to sufficient blood supply. Therefore, the aim of the present study was to investigate the influence of osteogenic stimulation and treatment with VEGF on new bone formation and neovascularization in hMSC-loaded cancellous bone scaffolds in vivo.

**Methods:**

Cubic scaffolds were seeded with hMSC and either cultured in stem cell medium or osteogenic stimulation medium. One osteogenically stimulated group was additionally treated with 0.8 μg VEGF prior to subcutaneous implantation in athymic mice. After 2 and 12 weeks in vivo, constructs and selected organs were harvested for histological and molecular analysis.

**Results:**

Histological analysis revealed similar vascularization of the constructs with and without VEGF treatment and absence of new bone formation in any group. Human DNA was detected in all inoculated scaffolds, but a significant decrease in cells was observed after 2 weeks with no further decrease after 12 weeks in vivo.

**Conclusion:**

Under the chosen conditions, osteogenic stimulation and treatment with VEGF does not have any influence on the new bone formation and neovascularization in hMSC-seeded cancellous bone scaffolds.

**Electronic supplementary material:**

The online version of this article (doi:10.1186/1471-2474-15-350) contains supplementary material, which is available to authorized users.

## Background

Large bone defects caused by trauma, tumor or infection still represent a major problem in reconstructive surgery. Tissue engineering approaches for reconstruction of such bone defects are appealing, but to date technically insufficient. In this context, one major problem – the blood supply during the first days in vivo – remains unsolved. Since the rate of vessel ingrowth into a cell-loaded scaffold is hypothesized to be less than one millimeter per day, a considerable period is needed to provide sufficient blood supply and delivery of substrates such as glucose and oxygen to inner parts of the engineered scaffold [1, 2]. Likewise, removal of waste products such as carbon dioxide and lactate via the bloodstream is delayed until a mature vascular system is established. Since bone represents a metabolically active tissue, the insufficient blood supply during the first days in vivo might compromise cell survival and hence lead to failure of the graft integration [2]. Therefore, various tissue engineering strategies have been developed to address this problem.

One promising approach is the use of growth factors such as vascular endothelial growth factor (VEGF), which plays an important role in inducing neovascularization and bone healing [3]. Kaigler et al. reported on significantly increased vascular perfusion and bone formation in irradiated osseous defects using VEGF-releasing polymer scaffolds [4]. Beside the improved blood supply, this phenomenon could also be ascribed to the VEGF-triggered bilateral communication between endothelial and osteogenic cell lineages leading to a broad proliferation of endothelial cells and differentiation of osteogenic progenitor cells into osteoblasts [5, 6].

The ability of hMSCs for differentiation along several cell lineages is well known and depends on the tissue source, whereas a substantial loss of their multi-potent properties was observed during culture in vitro [7]. Although many preclinical animal models showed ectopic as well as orthotopic bone formation following implantation of 3D cell-loaded constructs, questions regarding the optimal osteogenic differentiation method for hMSCs as well as an effective initiation of osteogenesis in vivo have not been finally clarified.

Therefore, the aim of this study was to determine if VEGF treatment of osteogenically stimulated hMSCs loaded on cancellous bone scaffolds is capable of enhancing neovascularization and bone formation in an ectopic mouse model.

## Methods

### Scaffold loading and cultivation of human mesenchymal stem cells

Human mesenchymal stem cells (hMSC) (Cambrex, East Rutherford, USA), which were harvested and purified from bone marrow aspirates of one healthy donor were purchased (Lonza, Basel, Switzerland) and cultivated in mesenchymal stem cell growth medium (MSCGM) (Lonza, Basel, Switzerland). Fresh medium was supplied three times per week. When cell layers neared confluence, cells were detached using trypsin-EDTA. Cultures were maintained in a humidified atmosphere of 95% air with 5% CO_2_ at 37°C.

The day before loading, cubic solvent-preserved and irradiated bovine cancellous bone scaffolds (Tutobone, Tutogen Medical, Neunkirchen am Brand, Germany) with an edge length of 3 mm were preincubated for 24 hours in MSCGM [8]. A suspension of 1.1 × 10^6^ hMSCs (passage 4) in 660 μl medium was evenly applied to each scaffold. For maximum seeding efficiency, scaffolds were turned and the cell suspension was resuspended onto each construct every 20 minutes. After 6 hours, all scaffolds were transferred to well-plates for further incubation. The seeding efficiency was assessed by measuring the number of remaining cells within the supernatant suspension in each well.

Following seeding, the scaffolds were cultured for 14 days either in MSCGM or in case of osteogenic stimulation in Dulbecco’s Modified Eagle Medium (DMEM, Gibco, Invitrogen, Carlsbad, USA) containing 10% fetal bovine serum (FBS, Sigma-Aldrich, St. Louis, USA),

4 mM L-Glutamine, 100 nM Dexamethasone, 10 mM b-Glycerophosphate and 50 mM L-Ascorbic acid 2-phosphate. Medium was changed every second day throughout the entire culture period. Directly before subcutaneous implantation, 6 osteogenically stimulated cell-loaded scaffolds were additionally injected with 0.8 μg human VEGF (Peprotech, Rocky Hill, USA).

### Experimental design and surgical procedure

**Figure 1:**
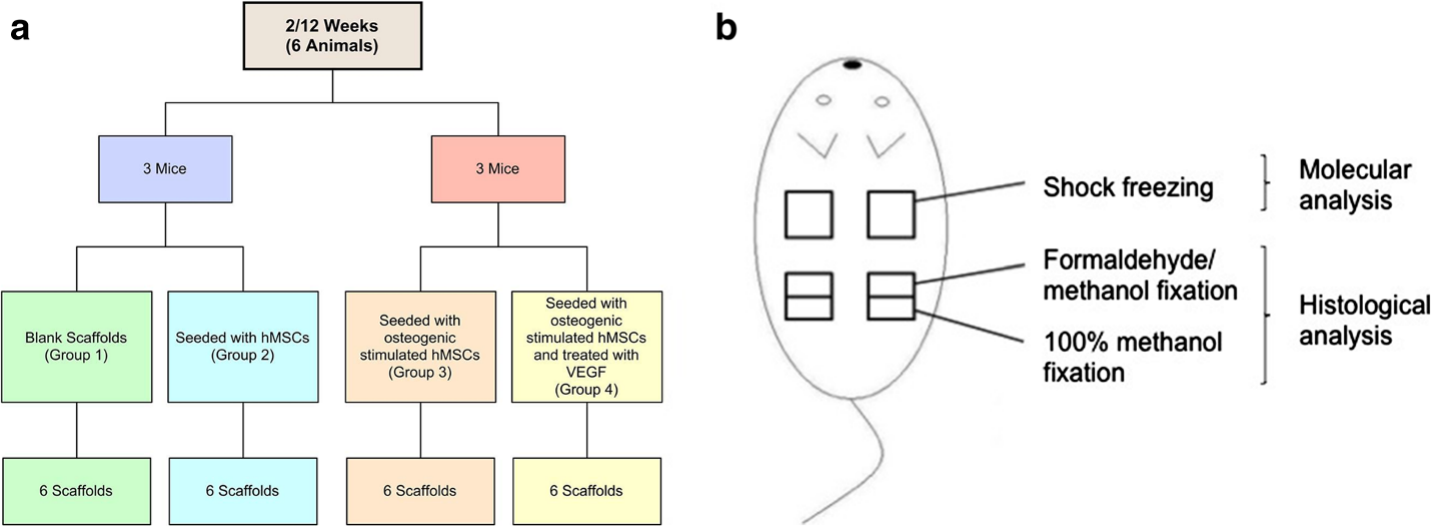
**Experimental setup.** In total 12 athymic nude mice ranging from six to eight weeks of age were used for this study **(a)**. According to the seeding and culture conditions, 4 groups were built: 1. blank scaffolds, 2. seeded with hMSCs, 3. seeded with osteogenically stimulated hMSCs, 4. seeded with osteogenically stimulated hMSCs and treated with VEGF. After 2 and 12 weeks, scaffolds were harvested including the surrounding tissue. One scaffold of each group was split into two parts **(b)**: one half was destined for decalcification the other for un-decalcified histological examination. The other scaffold of each group was shock frozen in fluid nitrogen and stored at -80°C until use for molecular analysis **(b)**.

In total 12 athymic nude mice ranging from six to eight weeks of age (nu/nu, Harlan Winkelmann, Rossdorf, Germany) with a live weight of 25-30 g were used for this study. Following intramuscular anaesthesia using a fentanyl-medetomidin-midazolam mixture (0,625 mg fetanyl, 0,125 mg medetomidin and 6,25 mg midazolam per kilogramm body weight), two subcutaneous paravertebral pouches were prepared by a blunt dissection. Within the first experimental setup, unstimulated empty scaffolds (n = 6, group 1) were implanted left paravertebrally, whereas scaffolds seeded with hMSCs (n = 6, group 2) were implanted on the right side. Scaffolds seeded with osteogenically stimulated hMSCs (n = 6, group 3) as well as an additional VEGF treatment (n = 6, group 4) were implanted in a second series paravertebrally to the right and left, respectively (Figure 1a). After implantation, pouches were sutured in order to prevent contact between implanted constructs.After 2 and 12 weeks, scaffolds were harvested including the surrounding tissue. One scaffold of each group was split into two parts; one half destined for decalcification was fixed in 100% methanol, while the other was put into a formaldehyde/methanol solution (formaldehyde 35%, methanol 100%, isotonic glucose 1% and phosphate buffer), both for 48 h (Figure 1b). The other scaffold of each group was shock frozen in fluid nitrogen and stored at -80°C until used for molecular analysis (Figure 1b). In addition, selected organs (blood, brain, heart, lung, liver, kidneys, spleen, testes, scaffold surrounding skin and muscle tissue) were harvested, snap frozen in liquid nitrogen and stored at -80°C until further use.

This study adhered to the ARRIVE guidelines, was approved by the “Government of Upper Bavaria” and all animals were handled according to the LMU guidelines (Ludwig-Maximilians-University of Munich) for the care and use of laboratory animals.

### Histological analysis

Scaffolds fixed in the formaldehyde/methanol solution were dehydrated in a graded series of ethanol over 5 days and afterwards embedded into methyl methacrylate (MMA). Next, 4 μm thick sections were collected from each scaffold by using a Polycut microtome (Reichert-Jung, Heidelberg, Germany). Sections were stained with Goldner’s Trichrome and Toluidine Blue.

Scaffolds fixed in methanol were decalcified with 5% EDTA/PBS solution over 4 weeks. The solution was changed every third day. Sections of 12 μm thickness were obtained by using a cryomicrotome (Microm, Walldorf, Germany) and then stained with Hematoxylin and Eosine.

All sections were investigated using a light microscope (Axioskop, Zeiss, Jena, Germany) and graded by 3 independent observers for the presence and extent of: i) granulation tissue, ii) necrosis, iii) fat cells, iv) neovascularization and v) foreign body giant cells. The grading scale ranged from 1 (tissue extent 1-20% of the scaffold/single cells in the border areas) to 5 (tissue extent 80-100%/lots of cells in all areas) according to the established method by van Gaalen [9].

### Extraction of genomic DNA

The isolation of DNA from the scaffolds was performed with DNeasy blood and tissue kit (Qiagen, Hilden, Germany) according to the manufacturer’s instructions. In brief, harvested tissue samples were lysed with proteinase K (>600 mAU/ml). The DNA in the lysed samples was then absorbed on a silica matrix and cell debris was removed by washing with DNeasy washing buffer. Finally, the DNA was collected by elution in AE buffer (Qiagen, Hilden, Germany) and photometrically quantified.

### Semiquantitative polymerase chain reaction (PCR)

Detection of human DNA within the scaffolds and various organs was performed by genomic PCR. An 850 bp fragment of the α-satellite region of the human chromosome 17 was amplified using primers as previously described in Becker et al. [10].

Each PCR reaction (end volume of 50 μl) contained 250 ng of human genomic DNA, 200 μM of nucleotide mixture, 250 μM of each primer, 2.5 U Taq Polymerase and Q-solution (Qiagen, Hilden, Germany) supplemented with 15 nM MgCl_2_.

An initial denaturation of 3 min. at 94°C was followed by 40 cycles comprised of 30 sec. at 94°C, annealing at 58°C for 1 min. and for another 1 min. a polymerase reaction at 72°C. Finally, 10 minutes of polymerisation and an interruption of the reaction by cooling down to 4°C completed the cycles. Amplified DNA fragments (850 bp) were transferred onto 1.75% agarose gels and after the electrophoresis, they were stained with ethidium bromide and analysed using ultraviolet light.

Isolated DNA from hMSCs served as positive control while DNA extracted from murine fibroblasts served as negative control in each PCR run. To prove the PCR sensitivity, genomic DNA mixture containing DNA from murine fibroblasts and hMSCs in different ratios (1 × 10^6^ murine fibroblasts + 10 hMSCs and 1 × 10^6^ mouse fibroblasts + 1 hMSC) was also used in each PCR run.

### Quantitative PCR

Quantitative PCR was performed by using LightCycler technology (Roche, Mannheim, Germany). A specific human primer set was purchased from Search-LC (Search-LC, Heidelberg, Germany). The final volume of each PCR-reaction was 20 μl containing 10 μl of 5 ng genomic DNA, 10 μl of a PCR-Mix (6 μl PCR grade water, 2 μl of primers and 2 μl of LightCycler Fast Start Master SYBR Green I; Roche).

Each PCR cycle consisted of a 10 second denaturation at 95°C followed by annealing at 68°C for 10 sec. with a transition to 58°C in steps of 0.5°C per cycle. Elongation was performed at 72°C for 16 seconds during which fluorescence intensity was measured in each cycle. The temperature transition rate of all steps was 20°C per second. Serial dilution steps of human genomic DNA in 10 μl distilled water (60 ng, 6 ng, 0.6 ng, 0.06 ng, 0.006 ng, 0.0006 ng DNA) were used to generate standard curves. In every PCR, DNA from hMSCs was used as a positive control, while DNA from murine fibroblasts served as a negative control.

Finally, the number of cells on the scaffold after explantation was calculated by the measured amount of human DNA and the common presumption of a genomic DNA content of 6 pg in eukaryotic cells.

### Statistical analysis

Comparison analysis was performed with the quantitative PCR data using SPSS software (IBM, Armonk, USA) and the unpaired t-test. The results are shown as mean ± standard deviation. A p-value of ≤0.05 was considered statistically significant.

## Results

### Seeding efficiency

**Figure 2:**
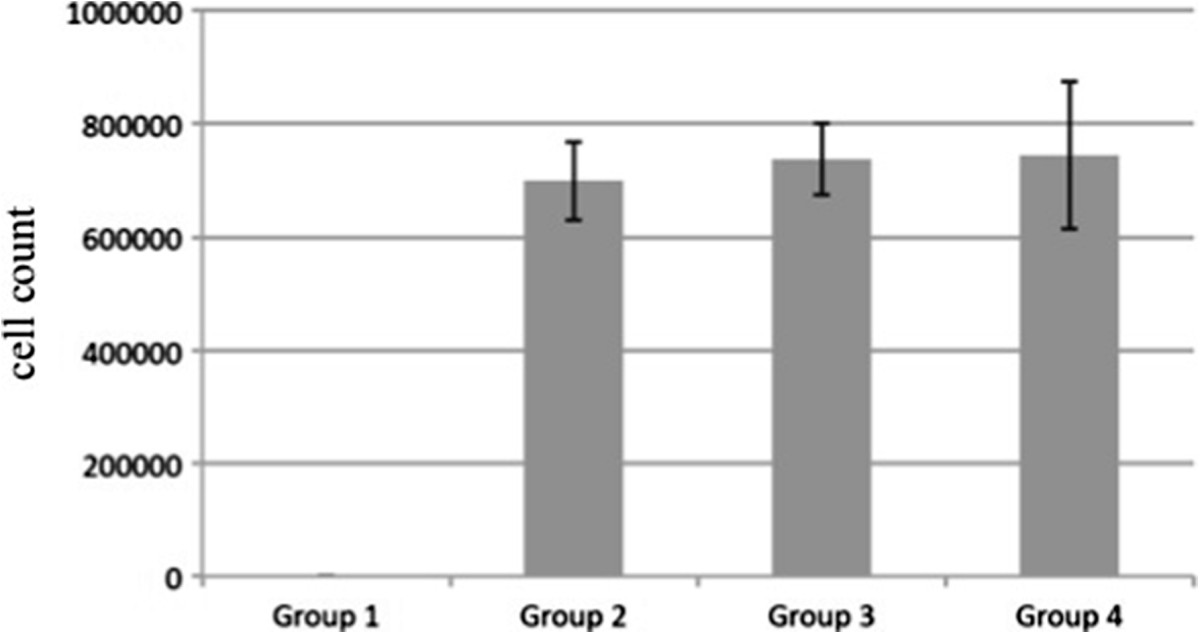
**Cell count after scaffold seeding with hMSCs.** From the original amount of 1.1 × 10^6^ hMSCs, a mean of 7.1 × 10^5^ cells adhered to the scaffold. Group 1: blank scaffolds (control); Group 2: seeded with hMSCs; Group 3: seeded with osteogenically stimulated hMSCs; Group 4: seeded with osteogenically stimulated hMSCs and treated with VEGF.

From the original amount of 1.1 × 10^6^ hMSCs applied onto the scaffolds, a mean of 7.1 × 10^5^ cells (median 7.0 × 10^5^ cells, range 4.1 × 10^5^ – 9.1 × 10^5^ cells) adhered to the scaffold (Figure 2). The mean seeding efficiency of all scaffolds was 64.8% (median 64.1%, range 41.7% – 83.3%). The statistical evaluation of seeding efficiencies did not show significant differences between the four experimental groups.

### Animal experiments

All animals showed good general conditions, dry wounds with no sign of irritation as well as appropriate behavior at the time of explantation. No animals died or suffered from diseases during the whole study duration prior to euthanasia.

### Histological findings

**Figure 3:**
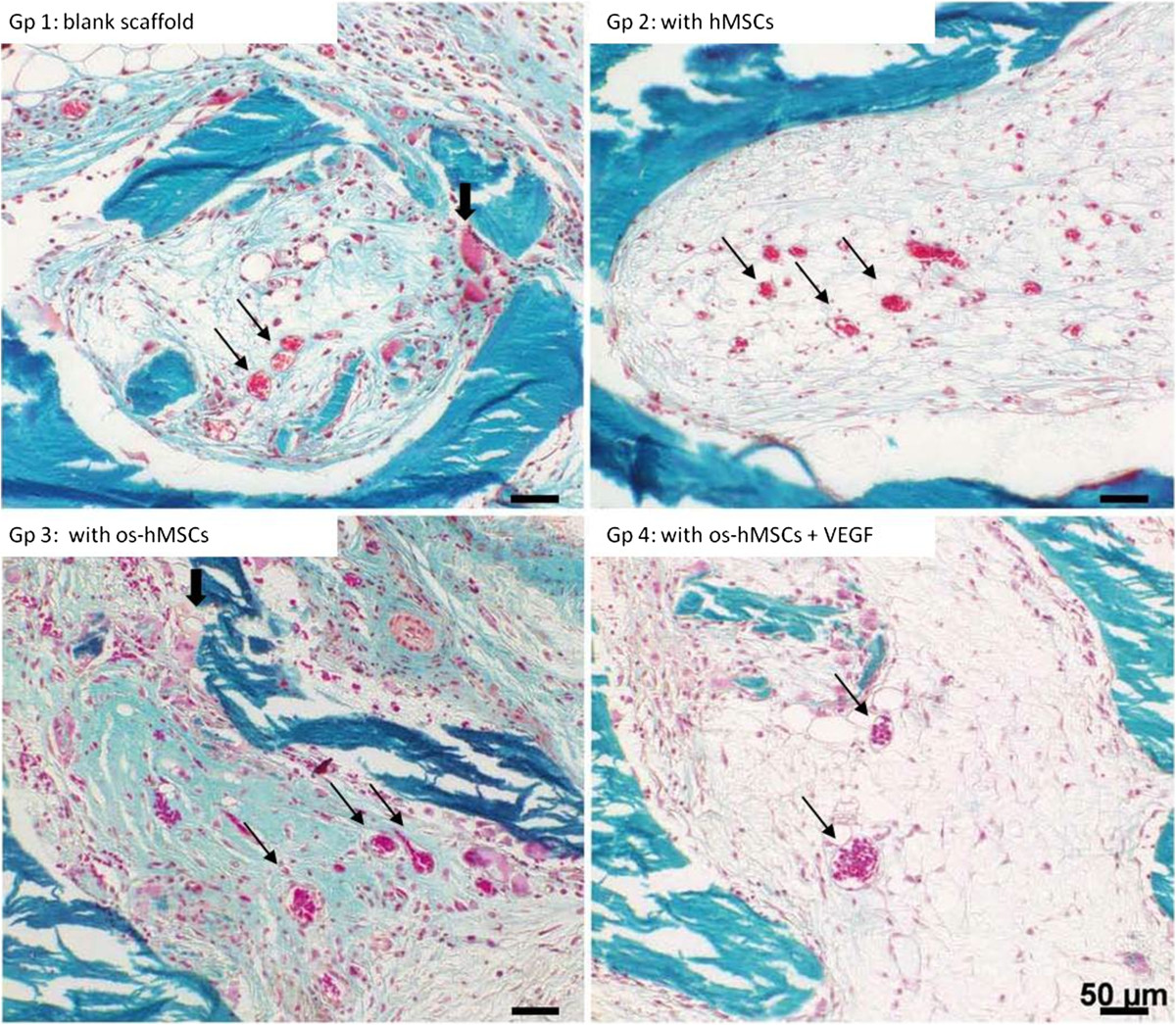
**Vascularization after 2 weeks.** Capillaries and small vessels (thin arrows) were detected in all groups after 2 weeks of in vivo implantation. In addition, multinucleated giant cells were observed on the scaffold surface (thick arrows). [Gp = Group, os-hMSCs = osteogenically stimulated hMSCs] Goldner’s trichrome: scale bars: 50 μms.

**Figure 4:**
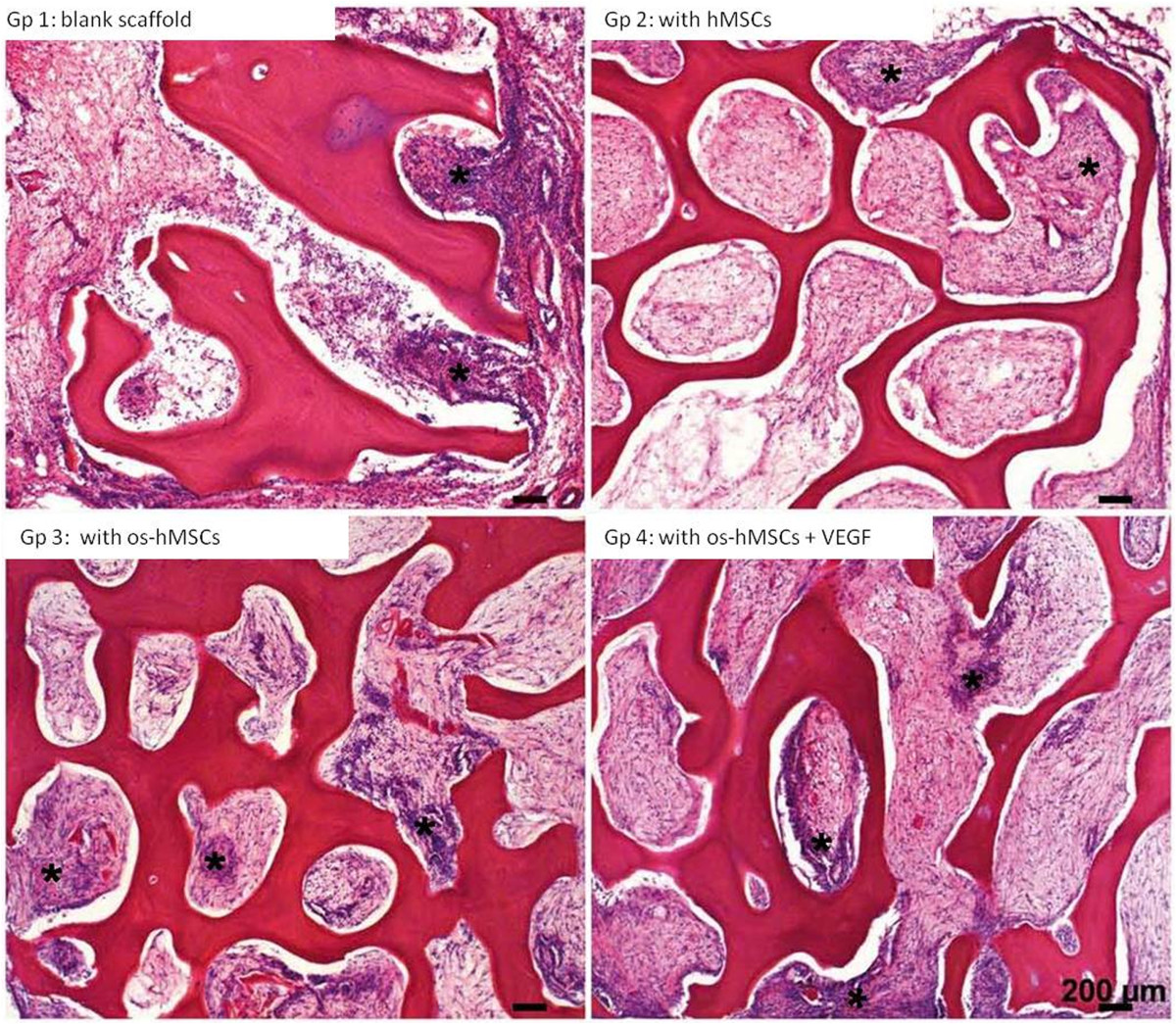
**Tissue after 2 weeks.** Granulation tissue (*) was observed in all groups after 2 weeks in vivo. [Gp = Group, os-hMSCs = osteogenically stimulated hMSCs] Hematoxylin-Eosin: scale bars: 200 μms.

**Figure 5:**
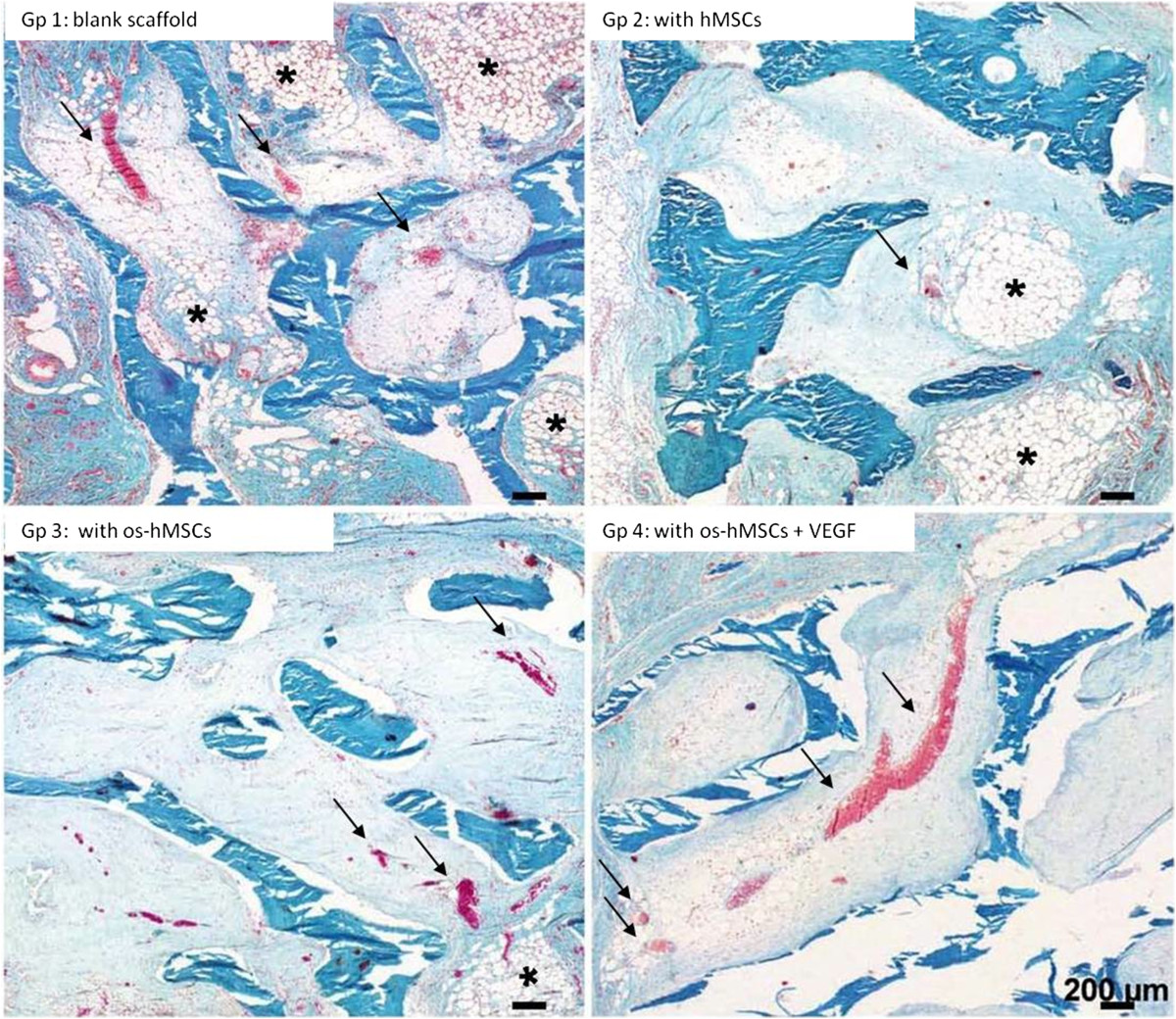
**Vascularization after 12 weeks.** After 12 weeks, further ingrowth of the surrounding tissue into the constructs occurred. Especially, a change in the vascularization (arrows) and the appearance of bigger fat cell aggregations (*) was noticed in all scaffolds. [Gp = Group, os-hMSCs = osteogenically stimulated hMSCs] Goldner’s trichrome: scale bars: 200 μms.

**Figure 6:**
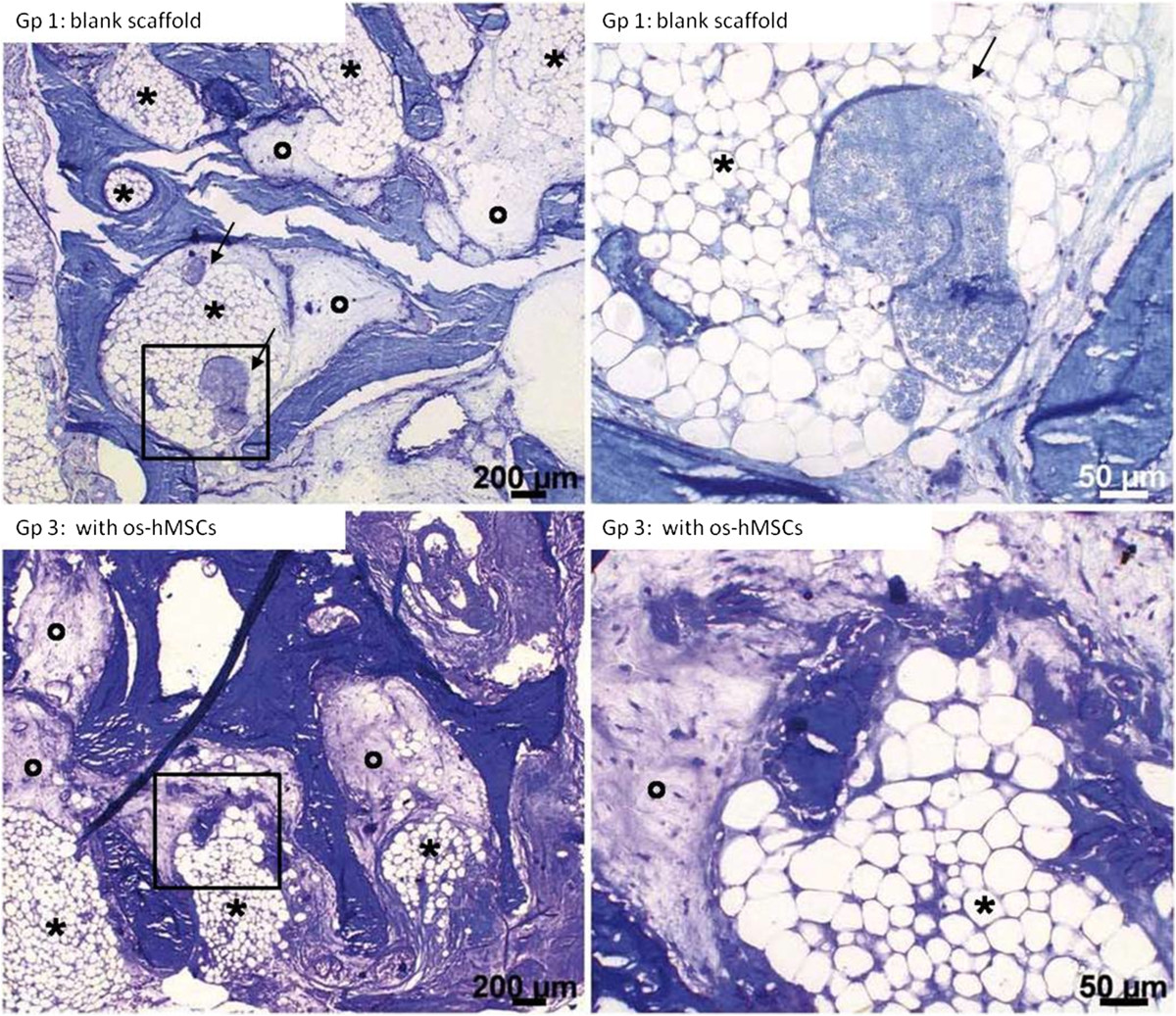
**Tissue after 12 weeks.** An increased expansion of fat cells (*) covering up to 60% of the scaffold surface and vessels (thin arrows) was found in all groups after 12 weeks in vivo implantation. Next to the fatty tissue, necrotic areas (°) were detectable in all groups. Pictures on the right side show the inserts in the left pictures. [Gp = Group, os-hMSCs = osteogenically stimulated hMSCs] Toluidine blue: scale bars: 200 μms (left), 50 μms (right).

After an implantation period of 2 weeks, an incipient ingrowth of fibrous- and granulation tissue as well as small vessels was observed in each scaffold. In all groups (1. unstimulated empty scaffolds, 2. inoculated with hMSCs, 3. inoculated with osteogenic stimulated hMSCs, 4. inoculated with osteogenically stimulated hMSCs and treated with VEGF), newly formed capillaries and small vessels were detected, lying evenly distributed mainly in the outer regions of the scaffold (Figure 3). Granulation tissue was also observed in all groups (Figure 4). Inner parts of the constructs contained scattered fat cells, inflammatory cells, and sometimes necrotic areas. Multinucleated giant cells were found in scaffolds from all groups. These cells were mostly aligned in groups of 2–4 along the edge of the scaffolds. Fat cells were observed within loose fibrous tissue, especially in outlying areas covering more than 20% of the scaffold surface. No apparent differences in the occurrence and distribution of necrotic areas were detected. Newly formed osteoid, bone- or cartilage tissue was not apparent after an implantation period of 2 weeks.In the 12 weeks group, further ingrowth of the surrounding tissue into the constructs occurred. Especially, a change in the vascularization and the appearance of bigger fat cell aggregations was noticeable in all scaffolds (Figure 5). Compared to the histological results after 2 weeks of implantation, a clear increase in size and amount of vessels was detected after 12 weeks in all groups. Capillaries, venules and arterioles were evenly distributed over all parts of the scaffolds. Furthermore, an increased expansion of fat cells was found in all groups, covering up to 60% of the scaffold surface in some cases (Figure 6). The highest extent of fatty tissue was observed in unstimulated empty (group 1) and inoculated scaffolds (group 2), while the lowest extent was found in osteogenically stimulated constructs (group 3). Next to the fatty tissue, flanking necrotic areas were detected. Multinucleated giant cells showed an inhomogeneous distribution pattern and were found in the scaffold periphery but also in central areas. After an implantation period of 12 weeks, neither newly formed osteoid nor bone or cartilage tissue was obtained.

No differences regarding vascularization or tissue reaction and formation were observed between the groups independent from the different culture and stimulation conditions.

### Semi-quantitative PCR

Human DNA was found in all inoculated scaffolds after explantation at both points in time. In blank scaffolds, no human DNA was detected. Moreover, we did not detect human DNA in any of the explanted organs as well as in the soft tissue surrounding the implanted scaffolds. Cell suspensions with hMSCs and murine cells served as controls allowing a detection of even 1 hMSC in 10^6^ murine cells.

### Quantitative PCR

**Figure 7:**
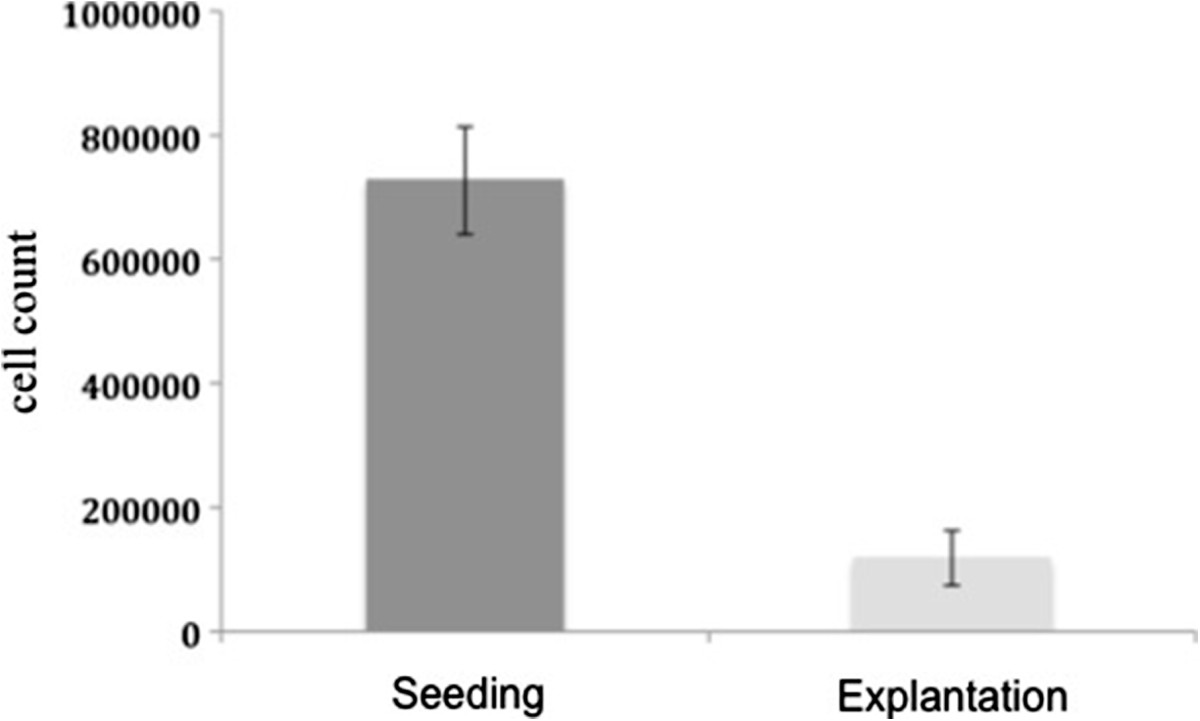
**Cells on the scaffold.** The number of cells on the scaffolds after explantation compared to the amount of initially seeded cells decreased in all groups.

**Figure 8:**
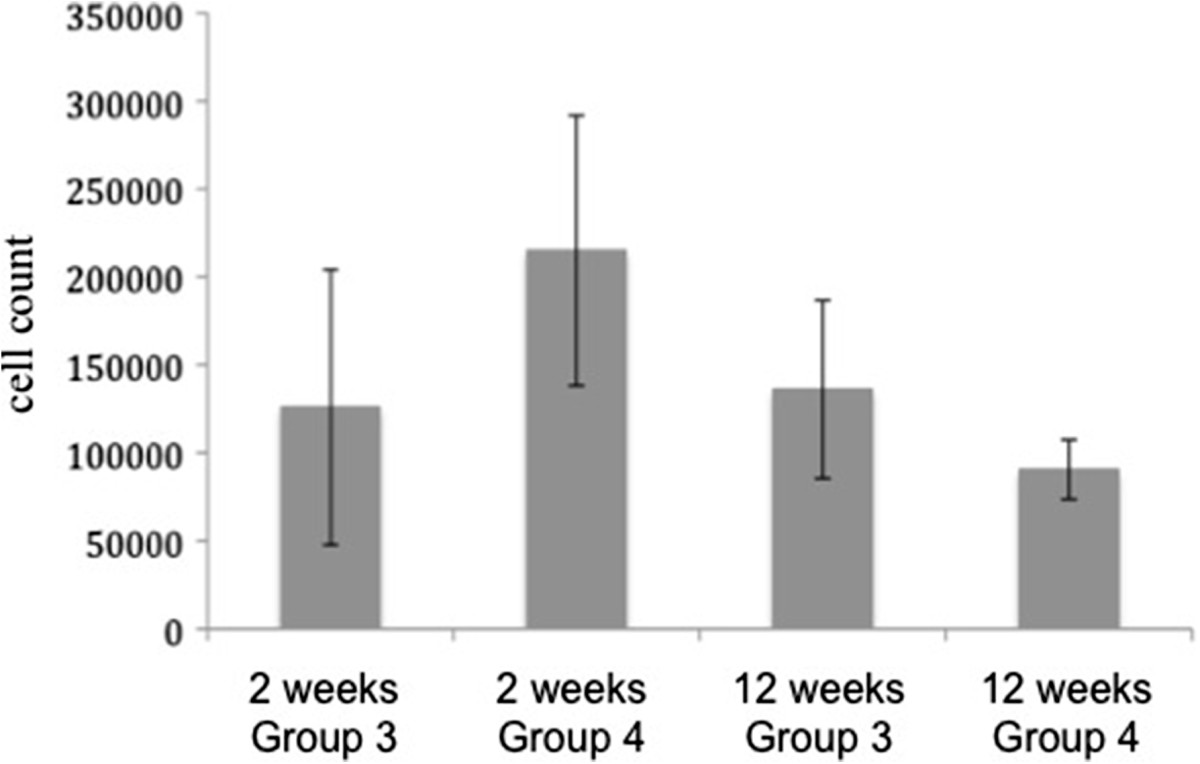
**Increase of cells in group 3.** Number of cells on the scaffolds after 2 and 12 weeks in vivo in group 3 (seeded with osteogenically stimulated hMSCs) and group 4 (seeded with osteogenically stimulated hMSCs and treated with VEGF).

With real time PCR, a significant decrease (p <0.001) of human DNA and accordingly the directly proportional cell numbers on all cell-seeded constructs (2 and 12 weeks in vivo) could be measured compared to the amount of initially seeded cells (Figure 7). After 12 weeks, a further decrease was detected in group 2 (inoculated) and 4 (inoculated with osteogenically stimulated hMSCs and VEGF treatment). Only for group 3 (seeded with osteogenically stimulated hMSCs), a slight increase of the cell number was observed after 12 weeks compared to 2 weeks (Figure 8). We identified higher cell numbers in the scaffolds with additional VEGF (group 4) after 2 weeks in comparison to just osteogenically stimulated scaffolds (group 3). After an implantation time of 12 weeks, group 4 showed inferior cell numbers. However, the above-mentioned differences were statistically not significant.

## Discussion

In this study, we aimed to evaluate the influence of osteogenic stimulation and application of vascular endothelial growth factor (VEGF) on bone formation in hMSC-seeded cancellous bone scaffolds after 2 and 12 weeks of subcutaneous implantation. During the implantation period, an ingrowth of surrounding host tissue was observed in all groups. Migration of human cells to host organs after xenogenic implantation was not seen in any of the animals, thus confirming previous results of our group [8]. Human DNA was detected in explanted scaffolds at all points in time, but a significant decrease in cells within the scaffolds could be measured in vivo compared to the amount of initially seeded cells. Neither bone formation nor higher amounts of neovascularization was observed. According to the journal guidelines ARRIVE check list for this study has been provided as Additional file 1.

### hMSC cultivation

The high amount of mesenchymal stem cells (hMSCs) within the commercially available cells used in this study was proven and described previously [11]. However, authors reporting on successful in vivo regeneration of bone often used BMSCs (Bone Marrow Stroma Cells) gained from bone marrow containing quantitatively more pre-differentiated progenitor cells than hMSCs [12, 13]. The presence of such progenitor cells, which can be expanded in vitro by osteogenic stimulation, is crucial for ectopic bone formation [14, 15]. In this context, additional contribution of BMP-2 and FGF-2 should be taken into account since an increased osteoblastic differentiation in vitro might have positive effects on ectopic bone formation in vivo [16].

The lack of newly formed bone could also be attributed to the static cultivation procedure. Since an ideal nutrient supply plays a major role during the initiation and maintenance of cell differentiation processes in vitro, a dynamic cultivation could be beneficial. Dynamic compared to static cultivation of osteogenically stimulated cell matrix constructs was shown to enhance the formation of an extracellular matrix in vitro which facilitates more new bone formation in vivo [17]. Additionally, the differentiation of osteogenically stimulated cells along the osteoblastic cascade is positively influenced by cytomechanical forces actuated within dynamic culture tanks [18]. In this context, various methods to stimulate cells with mechanical forces were reported including tensile stress, compressive stress, shear stress, vibration and magnetic stimuli [19].

A decrease of oxygen concentration towards the center of the scaffold was shown under static cultivation, whereas the oxygen concentration of dynamically cultured constructs did not fall below 4% after 5 days [20]. These data reinforce the assumption that a considerable number of cells probably died during static cultivation in vitro due to a decreasing oxygen gradient towards the inner parts of the construct. This could be one reason for our results which show a significant decrease of cell numbers after 2 weeks in vivo but no further significant decrease after 12 weeks.

### Vasculature

Taking into consideration that capillaries were predominantly located in the outer scaffold areas after 2 weeks in vivo, a further reason for the considerable decrease of hMSCs might be the lack of a sufficient vascular supply [8]. In our opinion, physical prerequisites regarding the diffusion threshold of 200 μm - 3.5 mm were met by the use of small cubic scaffolds with an edge length of 3 mm [21, 22]. However, considering the obtained results, cell death must be assumed at least for inner cell layers within the scaffold. Likewise, Potier et al. reported on broad cell death in vitro after a maximum of 5 days when the cells were exposed to hypoxia and deprivation of serum [23]. In contrast, maintaining constant or preconditioning levels of hypoxia was shown to improve the osteogenic potential of hMSCs and possibly preserve the stem cell character of hMSCs in vitro [24]. The fact that even in the VEGF group sufficient vascular supply was not initiated and cell survival was not achieved could be attributed to the biological instability of VEGF in vivo as well as the short stimulation time [3]. This shortcoming could be overcome by the use of novel scaffolds binding VEGF by heparin crosslinking and thus, achieving localized and sustained delivery of this growth factor [25]. Consistently, improved angiogenesis after subcutaneous implantation of heparinized scaffolds loaded with VEGF was observed [3]. Further improvements could be achieved by developing prevascularized cell-matrix-constructs providing a sufficient nutrient supply at early stages after implantation [26]. Prevascularized constructs, allowing an early functioning metabolic cycle, were shown to improve the survival rate of osteoblasts in vivo compared to non-prevascularized constructs [27].

### Tissue

The initiation of inflammatory processes by implantation of biological or synthetic materials is evident [8, 28, 29]. Essential parameters for the character and value of biological reaction are material properties and quality of the implanted foreign body [30]. Therefore, surface enlargement by means of interconnecting pores could be responsible for an increased invasion of reactive cells leading to foreign body reactions even in immunodeficient, athymic mice [31]. This might affect the survival of cells as a decrease of pH and release of degradation enzymes lead to changes in homoeostasis [30]. If and to which extent phagocytosis of human mesenchymal stem cells could take place in the course of such foreign body reactions remains unclear [32, 33].

Development of granulation tissue was observable in all groups after 2 weeks in vivo. We suggest that this phenomenon is to be explained by the physiological tissue chronology during foreign body reactions [30]. The production of e.g. TNFα or EGF by macrophages is responsible for the initiation of neovascularization and proliferation of fibroblasts resulting in the formation of granulation tissue, which is replaced by permanent tissue afterwards. Consistent with previous results, a regression of granulation tissue was observed after 12 weeks in all groups [8].

No bone formation was observed in any of the scaffolds indicating an insufficient amount of osteoprogenitor cells as well as vascular supply. Low osteogenic capacity of the ectopic implantation site as well as missing osteoinductive properties of the scaffold must be assumed as possible reasons for our results [15]. Therefore, implantation of the scaffolds in orthotopic sites might improve bone formation by chemotactic agents. However, Kruyt et al. have already demonstrated no significant difference in cell-based bone formation between ectopic and orthotopic implantation [34].

The study is limited, beside the abovementioned shortcomings, especially by a small sample size. Therefore, further studies are needed to clarify if VEGF is capable of stimulating murine cells. Alternatively, the use of co-cultures such as osteoblasts and endothelial cells could help to induce a sufficient blood supply in vivo and hence the new formation of bone.

## Conclusion

Under the chosen prerequisites, VEGF treatment of osteogenically stimulated hMSCs loaded on cancellous bone scaffolds is not sufficient to enhance neovascularization, bone formation and/or improved cell survival. Human DNA can be found in inoculated scaffolds even after 12 weeks in vivo but not in organs or the soft tissue surrounding following xenotransplantation.

## Electronic supplementary material

Additional file 1:ARRIVE check list.(DOCX 270 KB)

Below are the links to the authors’ original submitted files for images.

Authors’ original file for figure 1

Authors’ original file for figure 2

Authors’ original file for figure 3

Authors’ original file for figure 4

Authors’ original file for figure 5

Authors’ original file for figure 6

Authors’ original file for figure 7

Authors’ original file for figure 8

## Abbreviations

VEGF: Vascular Endothelial Growth Factor
hMSC: human Mesenchymal Stem Cells
EDTA: Ethylenediaminetetraacetic acid
CO2: Carbon dioxide
MSCGM: Mesenchymal Stem Cells Growth Medium
DMEM: Dubecco`s Modified Eagle Medium
FBS: Fetal Bovine Serum
MMA: Methyl Methacrylate
PBS: Phosphate Buffered Saline
DNA: Deoxyribonucleic acid
PCR: Polymerase Chain Reaction
SPSS: Statistical Package for the Social Sciences
BMSCs: Bone Marrow Stroma Cells
BMP-2: Bone Morphogenetic Protein-2
FGF-2: Fibroblast Growth Factor-2
pH: potential of hydrogen
TNFα: Tumor Necrosis Factor α
EGF: Epidermal Growth Factor.
