# Supplementary material for: Influence of osteogenic stimulation and VEGF treatment on in vivo bone formation in hMSC-seeded cancellous bone scaffolds
Source: BMC Musculoskelet Disord. 2014 Oct 16;15:350. doi: 10.1186/1471-2474-15-350 (PMC4216837; doi:10.1186/1471-2474-15-350)
Supplement: Supplementary file 1 — Additional file 1:ARRIVE check list.(DOCX 270 KB) [file 12891_2014_2289_MOESM1_ESM.docx]

ARRIVE checklist

Title

**1.) Title**: Influence of osteogenic stimulation and VEGF treatment on in vivo bone formation in hMSC seeded cancellous bone scaffolds.

Abstract

**2.) Abstract**: An accurate summary of the background, research objectives, key methods, principal findings and conclusion of the study was provided within the abstract.

Background:

Tissue engineering approaches for reconstruction of large bone defects are still technically immature especially in regard to sufficient blood supply. Therefore, the aim of the present study was to investigate the influence of osteogenic stimulation and treatment with VEGF on new bone formation and neovascularization in hMSC loaded cancellous bone scaffolds in vivo.

Methods:

Cubic scaffolds were seeded with hMSC and either cultured in stem cell medium or osteogenic stimulation medium. One osteogenically stimulated group was additionally treated with 0.8 μg VEGF prior to subcutaneous implantation in athymic mice. After 2 and 12 weeks in vivo, constructs and selected organs were harvested for histological and molecular analysis.

Results:

Histological analysis revealed similar vascularization of the constructs with and without VEGF treatment and absence of new bone formation in any group. Human DNA was detected in all inoculated scaffolds, but a significant decrease in cells was observed after 2 weeks with no further decrease after 12 weeks in vivo.

Conclusion:

Under the chosen conditions osteogenic stimulation and treatment with VEGF does not have any influence on the new bone formation and neovascularization in hMSC-seeded cancellous bone scaffolds.

Introduction

**3.)** **Background**: Scientific background as well as an explantation fort the experimental approach was included.

Likewise the reason for the use of an animal model was described.

**4.) Objectives**: The objective of this study was clearly stated: Aim of this study was to determine if VEGF treatment of osteogenically stimulated hMSCs loaded on cancellous bone scaffolds is capable of enhancing neovascularization and bone formation in an ectopic mouse model.

Methods

**5.) Ethical statement**: The study was approved by the “Government of upper Bavaria” whereas all animals were handled according to the LMU guidelines (Ludwig-Maximilians-University of Munich) for the care and use of laboratory animals. (manuscript page 5)

**6.) Study design**: Details of the study as the number of experiments and control groups were included. Additionally a flow chart (Figure 1,see below) was included illustrating the experimental setup. (manuscript page 5-8)


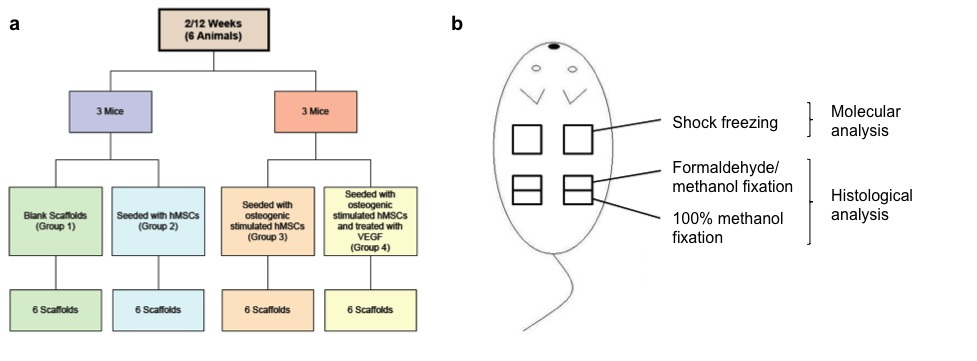


Figure 1 In total 12, six to eight weeks old, athymic nude mice were used for this study (a). According to the seeding and culture conditions 4 groups were built: 1. blank scaffolds, 2. seeded with hMSCs, 3. seeded with osteogenic stimulated hMSCs, 4. seeded with osteogenic stimulated hMSCs and treated with VEGF. After 2 and 12 weeks, scaffolds were harvested including the surrounding tissue. One scaffold of each group was split into two parts (b): one half was destined for decalcification the other for un-decalcified histological examination. The other scaffold of each group was shock-frozen in fluid nitrogen and stored at -80°C until used for molecular analysis (b).

**7.) Experimental procedure**: precise details of all procedures were provided including surgical techniques, anaesthesia/analgesia (0,625mg fetanyl, 0,125mg medetomidin and 6,25mg midazolam per kilogramm body weight) euthanasia as well as processing of samples. (manuscript page 5-8)

Briefly, cubic scaffolds were seeded with hMSC and either cultured in stem cell medium or osteogenic stimulation medium. One osteogenically stimulated group was additionally treated with 0.8 μg VEGF prior to subcutaneous implantation in athymic mice. After 2 and 12 weeks in vivo, constructs and selected organs were harvested for histological and molecular analysis.

**8.) Experimental animals**: A precise description of the used experimental animals such as source, age, number, live weight, genetic alteration and species was stated in the manuscript. (manuscript page 5)

**9.) Housing**: The housing conditions were adapted to the LMU guidelines (Ludwig-Maximilians-University of Munich) for the care and use of laboratory animals. Health conditions bofore and during the experiment were observed in a daily manner by a veterinary. (manuscript page 5)

**10.) Sample size**: The total number of animals was clearly stated in the manuscript. Additionally a flow chart (Figure 1) was added illustrating group size, differences between the groups, treatment conditions and time of experiments. As the study was a pilot project, a total of 12 mice were used in this experiment (see figure 1 and page 5). In total 6 scaffolds of each character were evaluated after 2 and another 6 after 12 weeks.

**11.) Allocating animals to experimental groups**: As all animals used in this experiments were comparable with regard to source, age, number, live weight, genetic alteration and species a randomisation was waived. All experiments were performed successively.

2 weeks experiments (explantation after 2 weeks): Within the first experimental setup (3 mice), unstimulated empty scaffolds (n=6, group 1) were implanted left paravertebrally, whereas scaffolds seeded with hMSCs (n=6, group 2) were implanted on the right side. Scaffolds seeded with osteogenically stimulated hMSCs (n=6, group 3) as well as an additional VEGF-treatment (n=6, group 4) were implanted in a second series (3 mice) paravertebrally to the right and left, respectively

12 weeks experiments (explantation after 12 weeks): analogue to 2 weeks experiments but scaffold explantation after 12 weeks.

**12.) Experimental outcome**: For histological outcome evaluation all histological sections were investigated for the presence and extent of granulation tissue, necrosis, fat cells, neovascularization and foreign body giant cells. The grading scale ranged from 1 (tissue extent 1-20% of the scaffold / single cells in the border areas) to 5 (tissue extent 80-100% / lots of cells in all areas) according to the established method by van Gaalen. Detection of human DNA within the scaffolds and various organs was performed by genomic PCR. Quantitative PCR was performed by using LightCycler technology. (manuscript page 6-8)

**13.) Statistical methods**: As the study was a pilot project and study groups rather small statistical analysis was conducted in a descriptive manner. All histological sections were investigated and graded by 3 independent observers (see 12.)

Comparison analysis for the quantitative PCR data was performed using SPSS software (IBM, Armonk, USA) and the unpaired t-test. The results are shown as mean ± standard deviation. A p-value of ≤ 0,05 was considered statistically significant. (manuscript page 6-8)

Results

**14.) baseline data**: As stated within the methods section only healthy mice were used for this study: In total 12 healthy, six to eight weeks old athymic nude mice (nu/nu, Harlan Winkelmann, Rossdorf, Germany) with a live weight of 25-30g were used. (manuscript page 5)

**15.) Numbers analyzed**: The number of mice used in this study (12 mice) was stated within the methods section as well as in Figure 1. All animals showed good general conditions, dry wounds with no sign of irritation as well as appropriate behavior at the time of explantation. No animals died or suffered from diseases during the whole study duration prior to euthanasia. All data were included.

**16.) Outcomes and estimations**: A precise description of all results either histological or molecular was stated within the manuscript. Briefly, histological analysis revealed similar vascularization of the constructs with and without VEGF treatment and the absence of new bone formation in any group. Human DNA was detected in all inoculated scaffolds, but a significant decrease in cells was observed after 2 weeks with no further decrease after 12 weeks in vivo. (manuscript page 8-10)


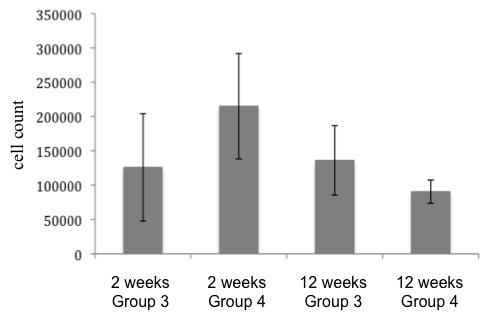


**Figure 2** Number of cells on the scaffolds after 2 and 12 weeks in vivo in group 3 (seeded with osteogenic stimulated hMSCs) and group 4 (seeded with osteogenic stimulated hMSCs and treated with VEGF).

17.) Adverse events: No adverse events were observed during the whole study duration. No modifications tp the experimental protocol were made.

Discussion

**18.) Interpretation/scientific implications**: A clear discussion of all relevant issues of this study was implemented into the manuscript including hMSC cultivation, development of vasculature and the absence of new bone formation. Limitations of the study as e.g. the small sample size were included at the end of the discussion section.

We furthermore stated, that additional studies will be needed to clarify if VEGF is capable of stimulating murine cells. Depending on the results further animal experiments using mice should be replaced by different experimental setups.

**19.) Generalisability/translation**: We conclude that under the chosen prerequisites VEGF treatment of osteogenically stimulated hMSCs loaded on cancellous bone scaffolds is not sufficient to enhance neovascularization, bone formation and/or improved cell survival. Human DNA can be found in inoculated scaffolds even after 12 weeks in vivo but not in organs or the soft tissue surrounding following xenotransplantation. However, our findings are not likely to translate to other species or systems.

**20.) Funding**: This work was supported by grants of the Bavarian Research Foundation (Collaboration for Tissue Engineering and Rapid Prototyping). We gratefully acknowledge Tutogen for providing the scaffolds (Tutobone). This work contains data from the theses of UL and FP and is published with permission of the medical faculty of the LMU Munich.

The authors declare that they have no competing interests.
