# Supplementary figures and images for: Influence of osteogenic stimulation and VEGF treatment on in vivo bone formation in hMSC-seeded cancellous bone scaffolds
Source: BMC Musculoskelet Disord. 2014 Oct 16;15:350. doi: 10.1186/1471-2474-15-350 (PMC4216837; doi:10.1186/1471-2474-15-350)

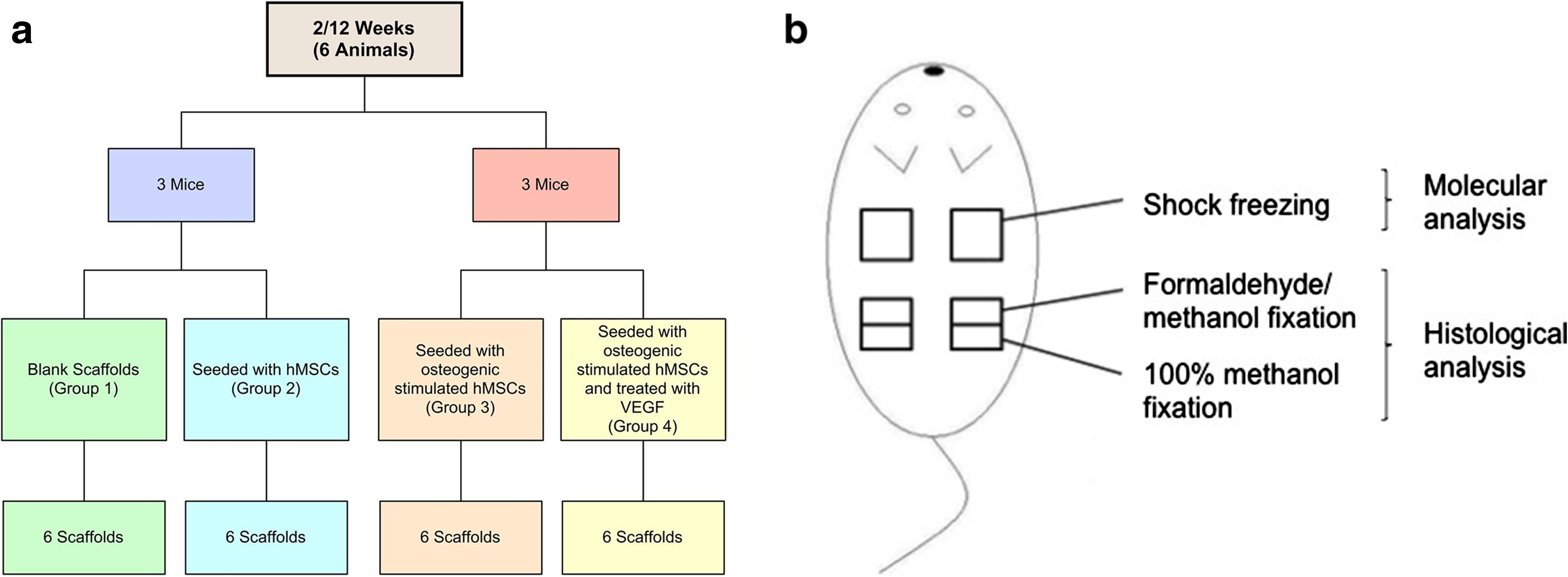

Supplement: Supplementary file 2 — Authors’ original file for figure 1 [file 12891_2014_2289_MOESM2_ESM.tiff]

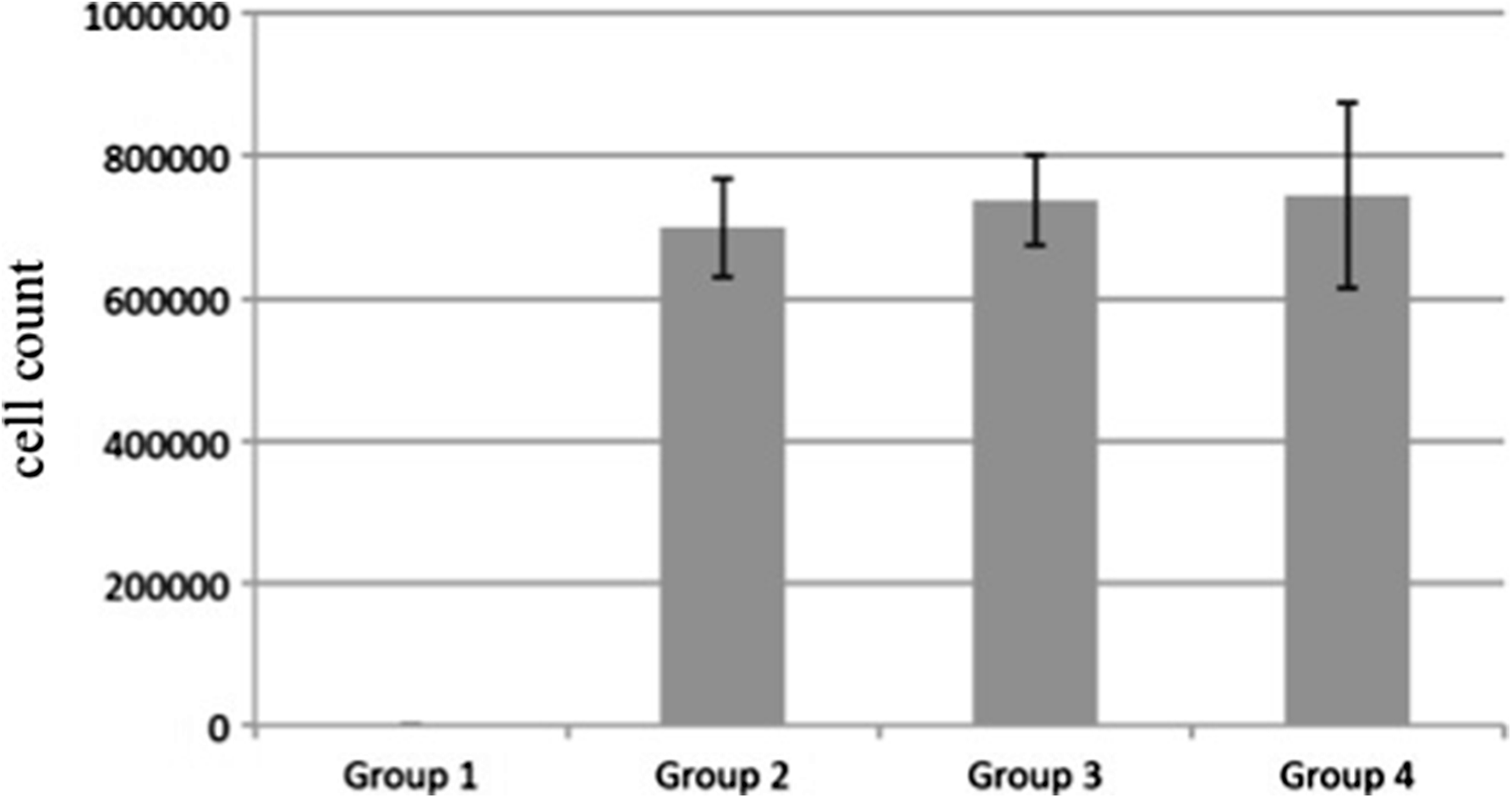

Supplement: Supplementary file 3 — Authors’ original file for figure 2 [file 12891_2014_2289_MOESM3_ESM.tif]

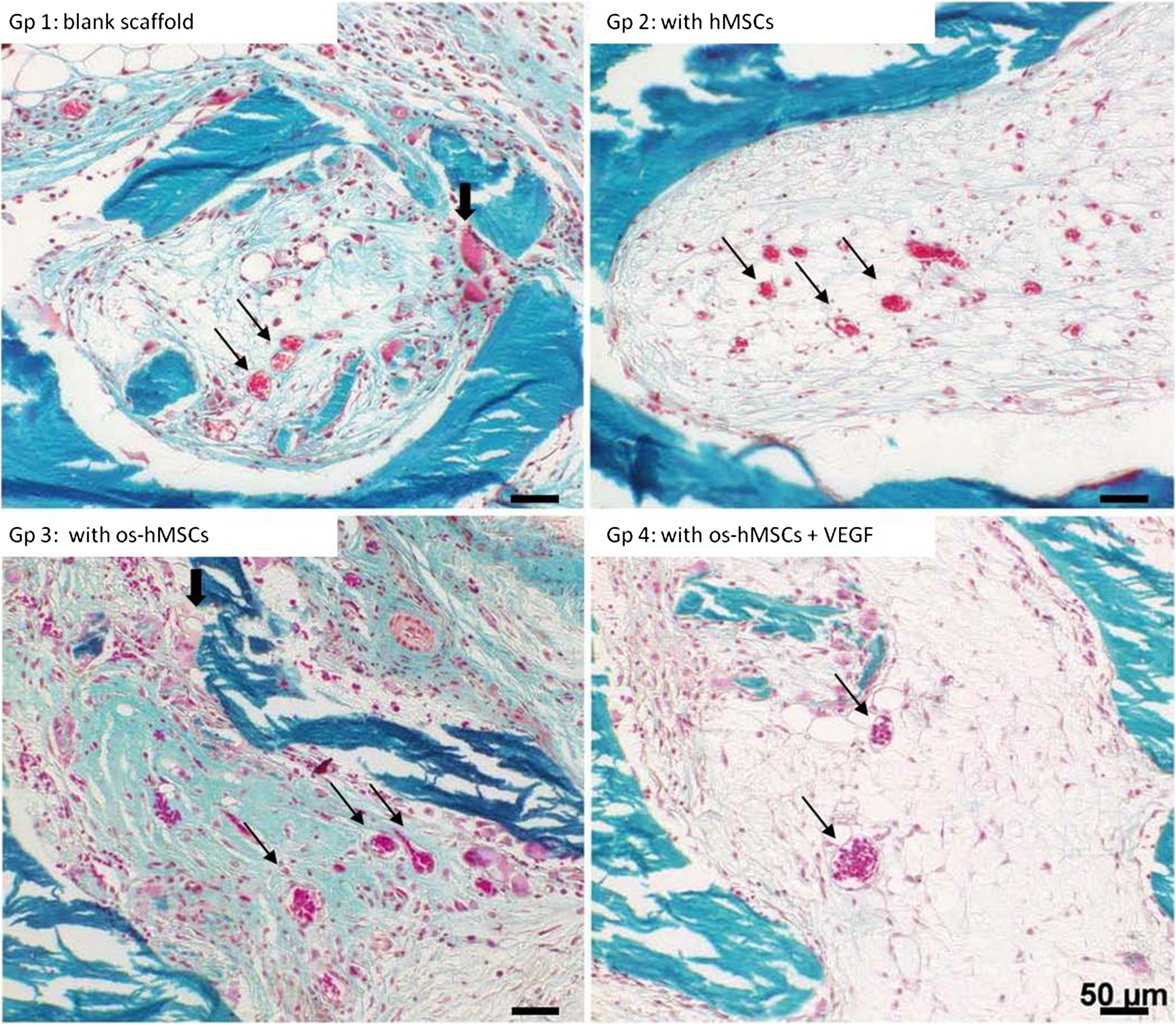

Supplement: Supplementary file 4 — Authors’ original file for figure 3 [file 12891_2014_2289_MOESM4_ESM.tif]

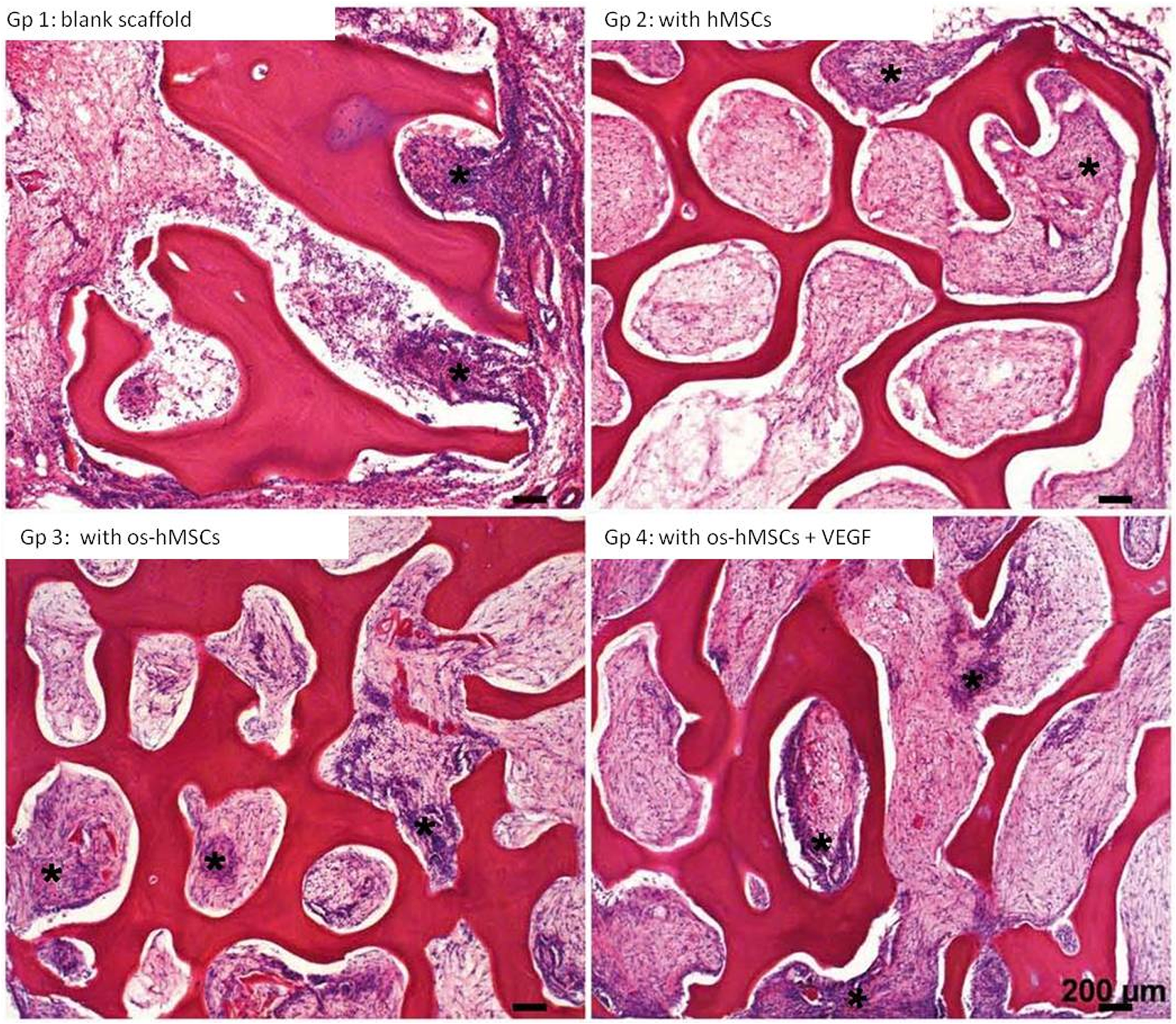

Supplement: Supplementary file 5 — Authors’ original file for figure 4 [file 12891_2014_2289_MOESM5_ESM.tiff]

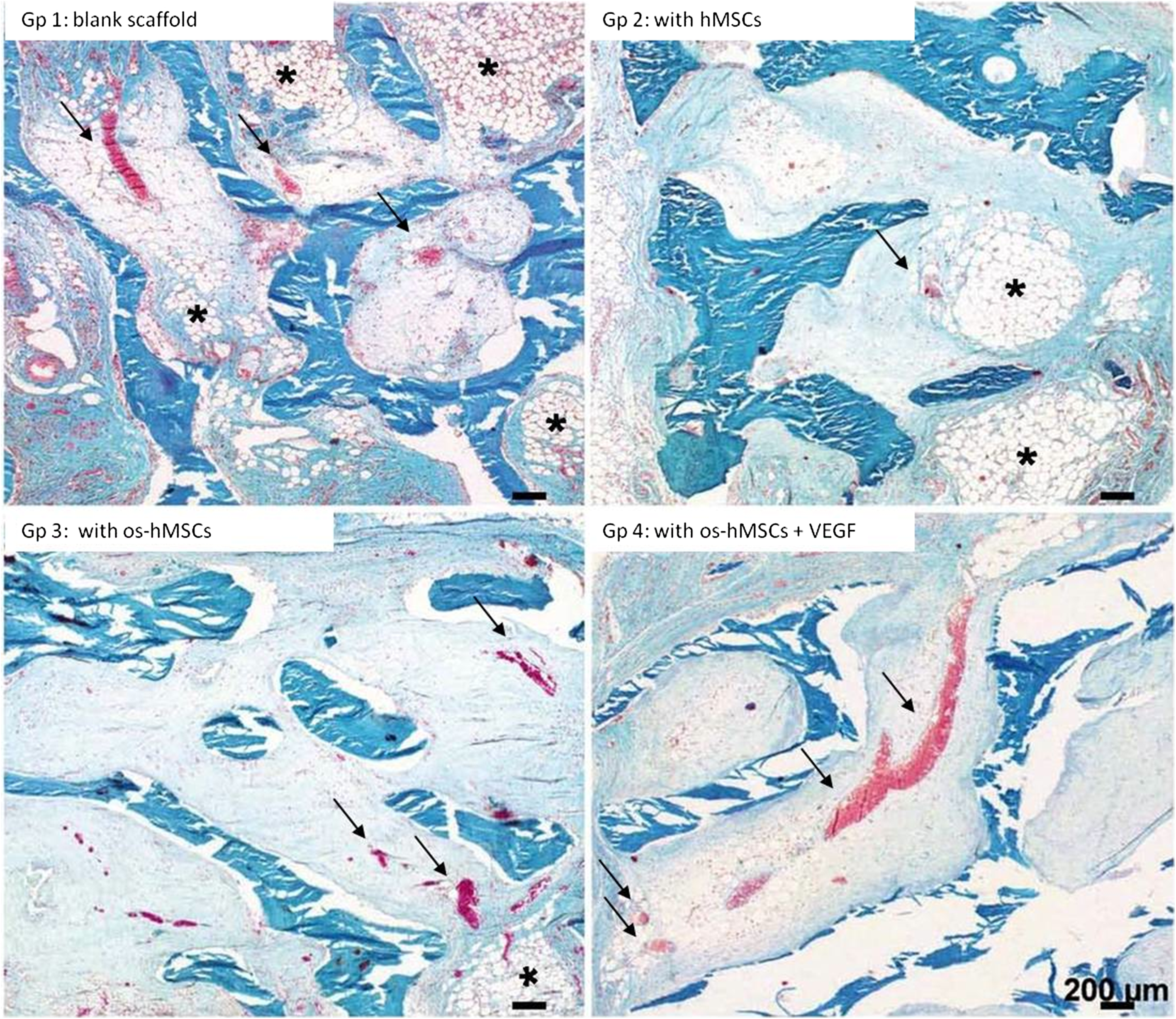

Supplement: Supplementary file 6 — Authors’ original file for figure 5 [file 12891_2014_2289_MOESM6_ESM.tif]

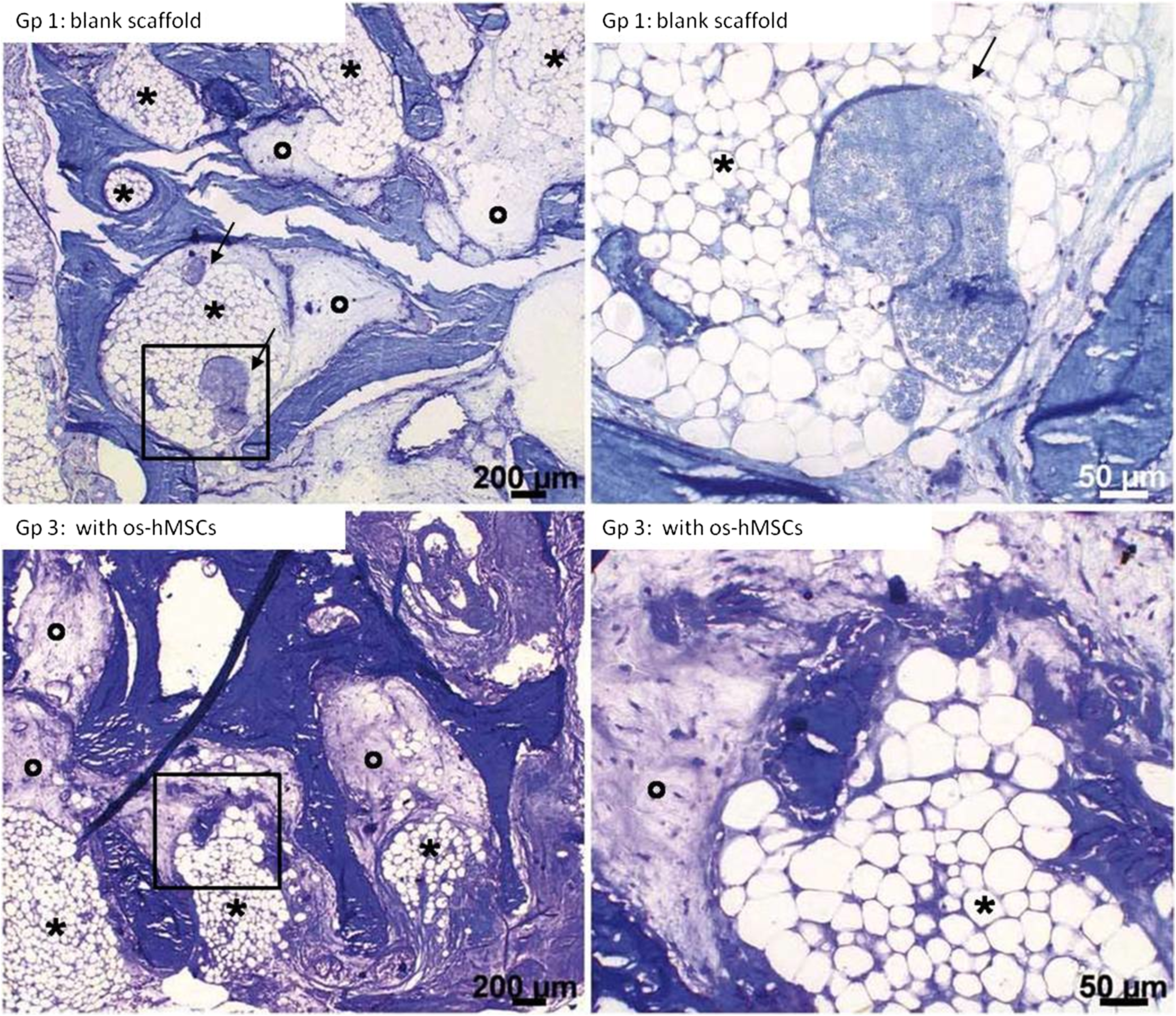

Supplement: Supplementary file 7 — Authors’ original file for figure 6 [file 12891_2014_2289_MOESM7_ESM.tif]

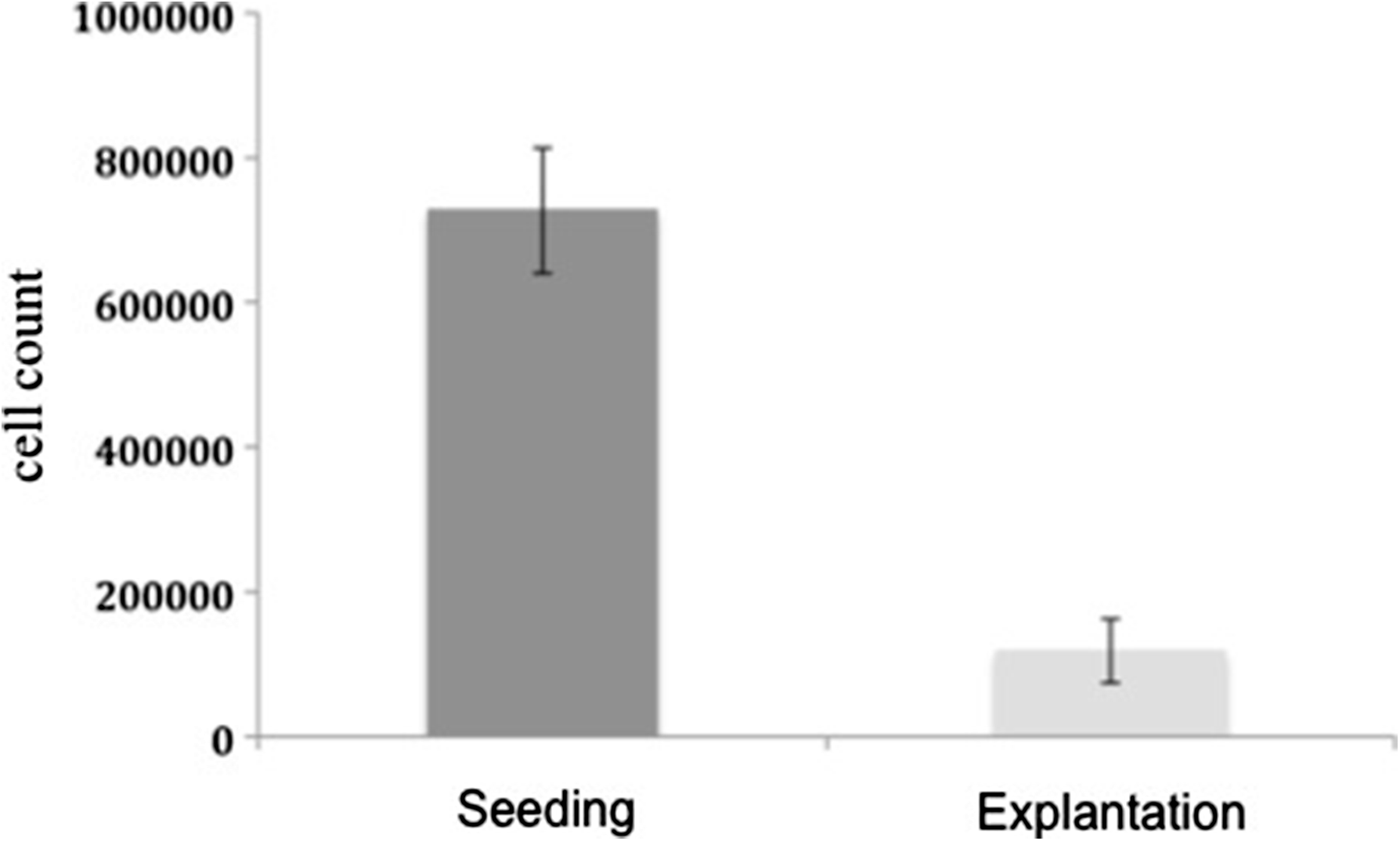

Supplement: Supplementary file 8 — Authors’ original file for figure 7 [file 12891_2014_2289_MOESM8_ESM.tif]

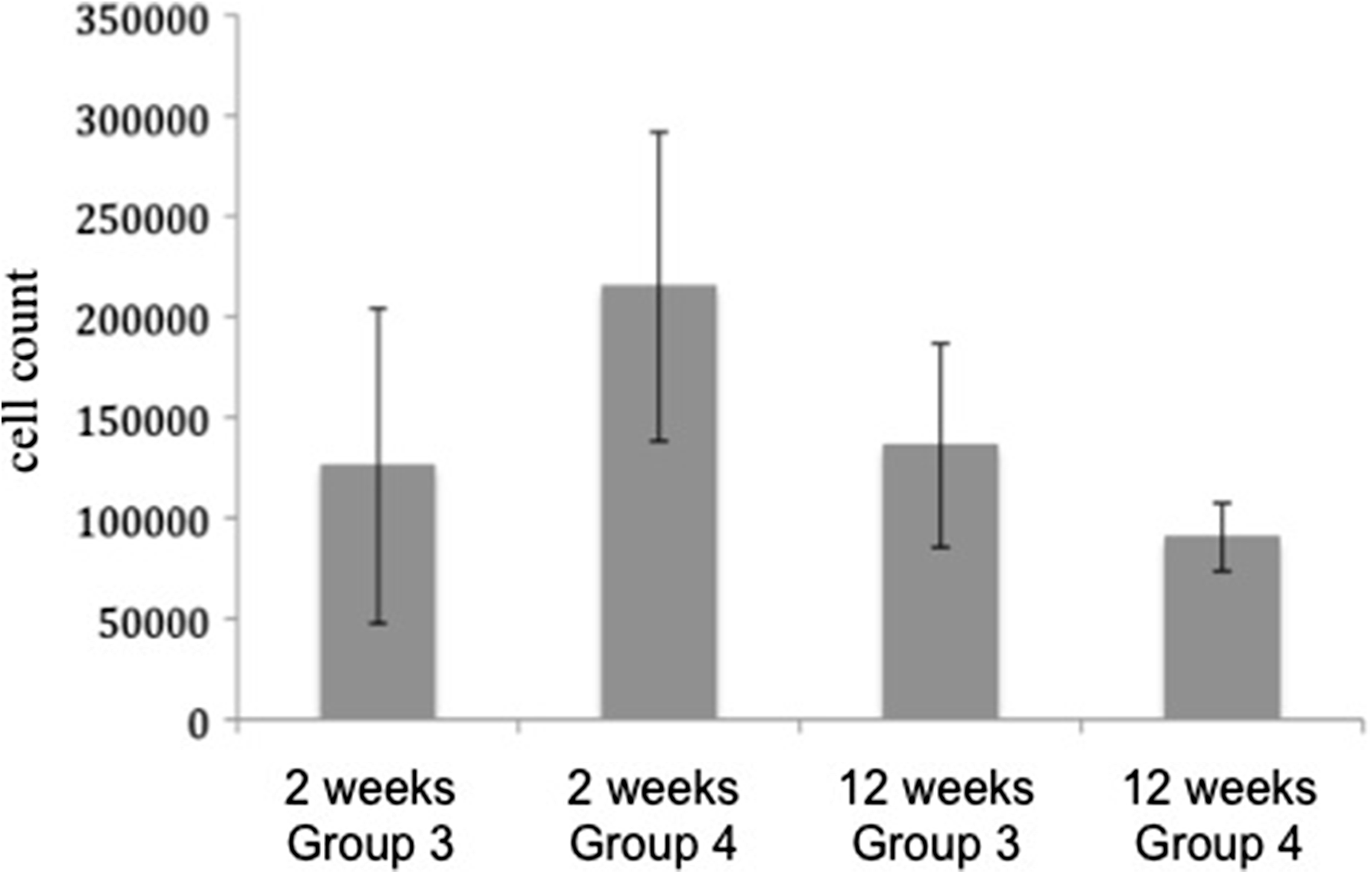

Supplement: Supplementary file 9 — Authors’ original file for figure 8 [file 12891_2014_2289_MOESM9_ESM.tif]
